# Reconstruction of Total Lower Eyelid Defects with the Temporoparietal Fascial Flap

**DOI:** 10.1155/2012/927260

**Published:** 2012-11-25

**Authors:** Simon R. Bababeygy, Anne R. Kao, Niels C. Kokot, Eli L. Chang

**Affiliations:** ^1^Doheny Eye Institute and the Department of Ophthalmology, Keck School of Medicine, University of Southern California, 1450 San Pablo Street, Los Angeles, CA 90033, USA; ^2^Division of Otolaryngology/Head and Neck Surgery, Keck School of Medicine, University of Southern California, 1500 San Pablo Street, Los Angeles, CA 90033, USA; ^3^Eye Physicians of Long Beach, 3325 Palo Verde Avenue, Suite 103, Long Beach, CA 90808, USA

## Abstract

*Purpose.* To describe the use of the temporoparietal fascial flap (TPF) in the reconstruction of extensive lower eyelid defects in a functioning eye. *Methods.* We present a surgical case report of a 73-year-old female with melanoma of the left lower eyelid. The lower eyelid was resected, and a composite nasal cartilage-mucosa graft, a skin graft, and a TPF were used to reconstruct the lower eyelid. *Results.* This achieved reconstruction of the lower eyelid with the protection of the eye and preservation of the visual system function. *Conclusion.* The TPF serves as a good option for reconstruction of the lower eyelid in a viable functioning globe.

## 1. Introduction

Reconstructing the eyelid in the setting of eyelid cancer excision presents a challenge for reconstructive success. This involves removal of important structures such as the periorbital skin, eyelashes, eyelid margins, upper and lower fornices, conjunctiva, tarsal plates, canthal ligaments, and canthal angles [1]. In certain circumstances, defects may extend well beyond the borders of the eyelid, requiring more extensive reconstruction. In such cases, standard reconstructive considerations for replacing the layers of the eyelid should be pursued like any other reconstructive case. However, replacement of the tissue surrounding the eyelid may require some form of volume augmentation. To help rectify this, a useful regional flap is the temporoparietal fascial flap (TPF).

The TPF is a thin, well-vascularized layer of moderately dense connective tissue lying beneath the subcutaneous tissues of the temporal scalp, just deep to the hair follicles. It forms a distinct fascial layer separated from the underlying deep temporal fascia by an avascular plane. The fascia is continuous with the superficial musculoaponeurotic system inferiorly and the galea aponeurotica superiorly. Preoperative examination by Doppler ultrasonography allows the course of the superficial temporal artery and its branches to be mapped out for reconstructive success. Here, we will describe a reported case of using the TPF in reconstruction in the setting of melanoma of the lower eyelid.

## 2. Case Report

### 2.1. Description

A 73-year-old Hispanic woman presented to clinic with a rapidly growing pigmented left lower eyelid lesion with extension of pigment to the bulbar conjunctiva. Biopsy results revealed malignant melanoma of the left lower lid, skin, and bulbar conjunctiva. The patient refused orbital exenteration and decided to undergo tumor excision surgery with the hope of achieving negative margins. She subsequently underwent a left lower eyelid resection with the use of a TPF reconstruction.

### 2.2. Surgical Approach

**Figure 1:**
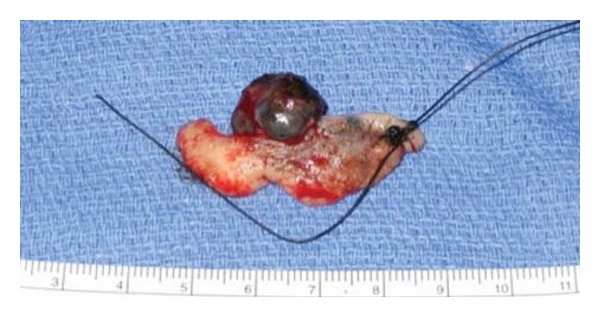
To remove the melanoma lesion, a full-thickness defect was created to excise the lower lid from the sidewall of the nose, down to the level of the inferior orbital rim.

A full-thickness defect was created using a no.15 blade to excise the lower lid from the sidewall of the nose, down to the level of the inferior orbital rim and across the entire horizontal length of the infraorbital rim and to the lateral canthus area. The eyelid and portion of the upper cheek was removed in toto (Figure 1).

**Figure 2:**
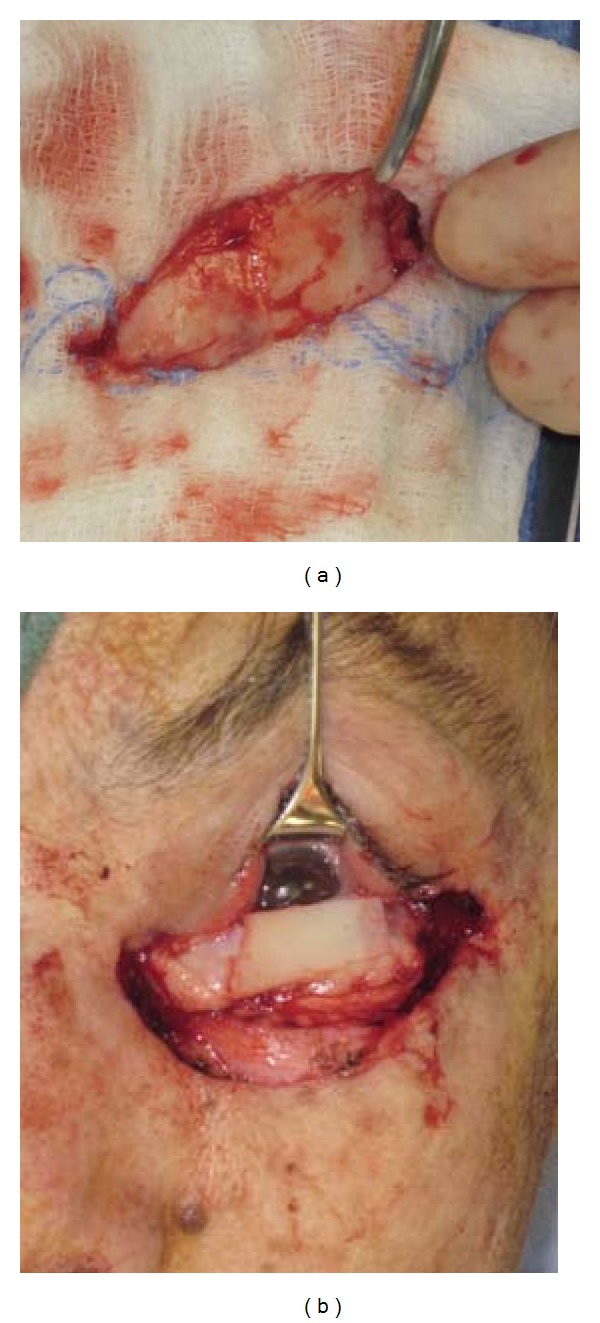
A 2 × 5 cm nasal septal cartilage graft was harvested from the left septum (a). The graft was placed into the defect as a tarsus with the mucosa facing the eyelid (b).

A 2 × 5 cm composite nasal cartilage-mucosa graft was harvested from the left septum of the patient and was placed into the defect as a tarsus with the mucosa facing the eyelid (Figures 2(a) and 2(b)). The fornix was first reformed by suturing the remnants of the bulbar conjunctiva to the sutures. The medial canthus was reformed by placing two sutures through the remnants of the medial canthal tendon. These sutures were then placed through the medial edge of the cartilage mucosal graft in a mattress fashion and then tied. Sutures were placed through the periosteum of the lateral orbital rim slightly above the original insertion site of the lateral orbital tubercle and used to secure the lateral edge of the composite graft.

**Figure 3:**
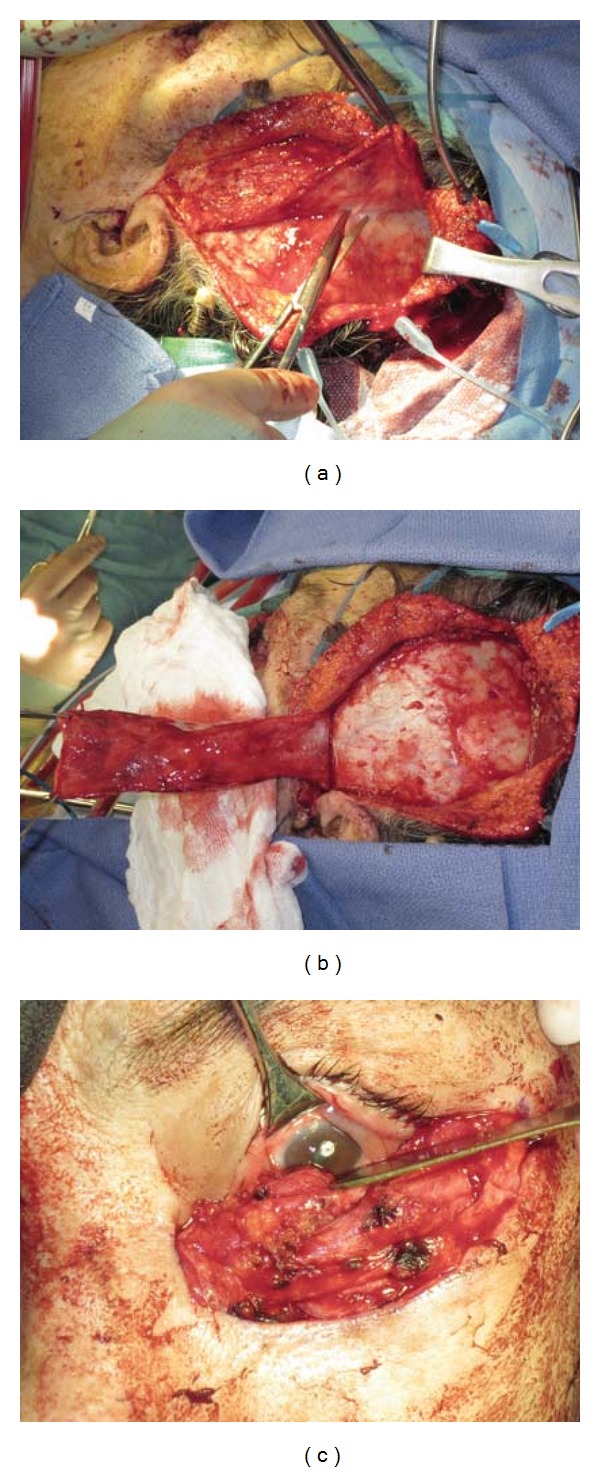
In a lateral quadrant, dissection of the TPF was carried down to the level of the periosteum (a). The fascial flap was then elevated off the underlying deep temporal fascia (b). At the level of the orbital rim, the flap was retrieved and pulled across the lower lid defect and sutured into the medial canthus area (c).

To begin the TPF, the superficial temporal artery was palpated approximately a centimeter anterior to the root of the helix. Local anesthesia with epinephrine was infiltrated along the course of the skin incision from the tragus of the ear along the course of a hemicoronal scalp incision. The incision was made to the level just deep to the hair follicles of the scalp. Dissection proceeded in the immediate subfollicular plan to expose the underlying superficial temporal artery and vein with their investing fascia. Bipolar cautery was used to prevent compromise of the flap vascularity. After exposing the artery and its fascia to approximately the midline of the head, the fascia was incised to generate an approximate 4-5 cm wide flap. In a lateral quadrant, dissection was carried down to the level of the periosteum. At this level, periosteum was reflected away from the bone exposing the underlying temporalis muscle and temporalis fascia. The fascial flap was then elevated off the underlying deep temporal fascia and pedicled entirely on the superficial temporal artery and vein (Figures 3(a) and 3(b)).

Using endoscopic visualization and endoscopic instruments, a tunnel in a plane deep to the TPF, just above the true temporalis fascia was created approximately 2 cm above the zygomatic arch to the level of the lateral orbital rim. At the level of the orbital rim, the flap was retrieved and pulled across the lower lid defect and sutured into the medial canthus area over the composite nasal cartilage-mucosa graft (Figure 3(c)). The TPF was then secured to the rest of the lower lid defect. At the superior edge, the TPF was secured to the tarsus mucosal graft, ensuring draping of the mucosa over the edge of the TPF. The TPF was then secured at the lateral canthus area by placing a vicryl suture through the periosteum of the lateral orbital rim, and then through the edge of the TPF. The inferior edge of the TPF was anchored to the remnants of the suborbicularis oculi fat pad using multiple bites of vicryl suture.

**Figure 4:**
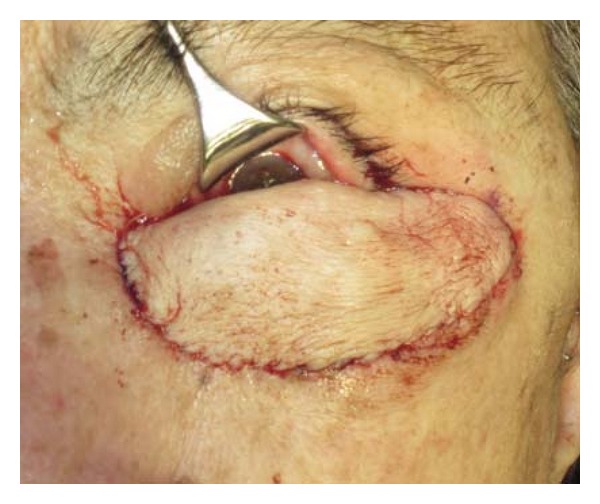
An elliptical skin graft approximately 5 × 8 cm was removed from the right underarm and was sutured over the TPF.

Once the TPF had been anchored into position overlying the defect, attention was then turned to the skin graft. An elliptical skin graft approximately 5 × 8 cm was removed from the forearm and was placed over the TPF and secured at the cardinal points using buried sutures (Figure 4). The remainder of the closure was completed using a combination of interrupted and running sutures. Great care was taken at the lid margin to ensure that mucosa from the composite graft was draped over the margin edge to prevent rubbing of keratinized skin against the globe. A pressure dressing was applied to the area to prevent hematoma formation. Postoperatively, the patient healed well, and her pain was well controlled. The flap was edematous in the immediate postoperative period, but the edema resolved, and the flap was successful.

## 3. Discussion

Reconstruction of the eyelid around the periorbital space could present as a challenge to reapproximate proper tissue planes to mimic the function of the eyelid without compromising the globe and visual system [1]. The periorbital space and eye socket have been reconstructed using grafts from dermal fat, cartilage, bone, muscles, or free flaps [2–6]. Recent reports of using the TPF have been reported in the reconstruction of anophthalmic orbit with a contracted eye socket [7–9] and after enucleation, exenteration [10, 11].

In our case, infiltration of melanoma into the left lower eyelid was the culprit necessitating tissue reconstruction in a viable eye with 20/20 visual acuity. However, irrespective of the cause of tissue loss, the fundamental goal of eyelid reconstruction remains constant: the protection of the eye and preservation of the visual system function.

The challenge in reconstruction of the lower eyelid is making sure that there is adequate vascular supply. This was easily achieved given that the TPF is well vascularized. As a robust flap with excellent vascular supply, the TPF is able to support a graft on both sides of the flap, allowing grafting of the cartilage on one side and skin on the other side. Additionally, there must be a smooth mucous membrane lining the inner surface of the eyelid to avoid ocular surface damage and provide lubrication to the eye. We were able to achieve this by placing the epithelial lining tissue of the nasal septal cartilage. Additionally, the septal cartilage provided structural stability, substituting the functional support provided by the tarsus. This helps provide stability and fixation to prevent eyelid malposition and contour defects. Given that there is loss of muscle function of the lower lid, it was important to reapproximate the tissues to a slightly higher axis while still providing clear central visual axis without compromising proper visual function. Doing so allows the upper eyelid to provide adequate function of appropriate closure of the eyelid for protection and lubrication.

 In conclusion, the technique discussed in this paper is limited by surgeon experience and tissue availability. Top priority is to protect the eye and secondarily to reconstruct the eyelid in a functional and aesthetically proper fashion. It is important that the tissue in contact with the surface of the globe must be lined with a mucosalized layer. Furthermore, the eyelid needs to have structural stability to avoid detrimental malpositions such as entropion or ectropion and prevent lagophthalmos or corneal exposure. The authors describe the use of the TPF as a good option for reconstruction of the lower eyelid in a viable functioning globe. Great precision and care must be taken in order to achieve a proper reconstruction using this method, providing an important solution for a difficult case.
